# Revolutionizing Pulmonary Diagnostics: A Narrative Review of Artificial Intelligence Applications in Lung Imaging

**DOI:** 10.7759/cureus.57657

**Published:** 2024-04-05

**Authors:** Arman Sindhu, Ulhas Jadhav, Babaji Ghewade, Jay Bhanushali, Pallavi Yadav

**Affiliations:** 1 Respiratory Medicine, Jawaharlal Nehru Medical College, Datta Meghe Institute of Higher Education and Research, Wardha, IND; 2 Obstetrics and Gynecology, Jawaharlal Nehru Medical College, Datta Meghe Institute of Higher Education and Research, Wardha, IND

**Keywords:** disease prognosis, healthcare, machine learning, lung imaging, pulmonary diagnostics, artificial intelligence

## Abstract

Artificial intelligence (AI) has emerged as a transformative force in healthcare, particularly in pulmonary diagnostics. This comprehensive review explores the impact of AI on revolutionizing lung imaging, focusing on its applications in detecting abnormalities, diagnosing pulmonary conditions, and predicting disease prognosis. We provide an overview of traditional pulmonary diagnostic methods and highlight the importance of accurate and efficient lung imaging for early intervention and improved patient outcomes. Through the lens of AI, we examine machine learning algorithms, deep learning techniques, and natural language processing for analyzing radiology reports. Case studies and examples showcase the successful implementation of AI in pulmonary diagnostics, alongside challenges faced and lessons learned. Finally, we discuss future directions, including integrating AI into clinical workflows, ethical considerations, and the need for further research and collaboration in this rapidly evolving field. This review underscores the transformative potential of AI in enhancing the accuracy, efficiency, and accessibility of pulmonary healthcare.

## Introduction and background

Pulmonary diagnostics encompass a range of medical procedures to assess the health and function of the respiratory system, particularly the lungs. These diagnostics include imaging techniques such as X-rays, computed tomography (CT) scans, and magnetic resonance imaging (MRI), as well as pulmonary function tests and bronchoscopy [1]. Accurate and efficient lung imaging is crucial for the early detection, diagnosis, and management of various pulmonary conditions, including lung cancer, pneumonia, chronic obstructive pulmonary disease (COPD), and interstitial lung diseases (ILDs). Timely and precise imaging helps clinicians make informed decisions about patient care, treatment planning, and monitoring disease progression [2].

Artificial intelligence (AI) has emerged as a transformative technology in healthcare, offering innovative solutions to improve diagnostics, treatment, and patient outcomes. In pulmonary imaging, AI algorithms and machine learning techniques have shown promising results in automating image analysis, detecting abnormalities, and predicting disease prognosis [3]. This review aims to comprehensively explore the applications of AI in revolutionizing pulmonary diagnostics, focusing on lung imaging. By synthesizing current research, case studies, and emerging trends, the review seeks to provide insights into the potential impact of AI on enhancing the accuracy, efficiency, and accessibility of pulmonary healthcare.

## Review

Traditional methods in pulmonary diagnostics

X-rays

X-rays represent a form of high-energy electromagnetic radiation extensively employed in medical imaging to generate images of internal bodily structures. By traversing the body, X-rays produce detailed images of tissues, organs, bones, and teeth, aiding healthcare professionals in diagnosing various conditions ranging from fractures and injuries to infections and anomalies. These images are crafted by employing external radiation to produce a negative portrayal wherein denser structures, such as bones, manifest as white, while softer tissues, like skin and muscle, register as dark gray [4,5]. X-ray technology is pivotal across various diagnostic procedures, including arteriograms, CT scans, and fluoroscopy. Despite their indispensable utility in medical diagnostics and material science due to their capacity to penetrate solid substances such as living tissues, it's imperative to acknowledge that X-rays constitute ionizing radiation. Exposure to elevated intensities of X-rays carries inherent health risks, encompassing DNA damage, cancer, burns, and radiation sickness. Consequently, the generation and utilization of X-rays are rigorously regulated by public health authorities to mitigate potential hazards [6-8].

CT Scans

CT scans are diagnostic imaging procedures that utilize ionizing radiation to generate detailed cross-sectional images of the body, encompassing internal organs, blood vessels, soft tissues, and bones [9,10]. These scans are pivotal tools in the detection, diagnosis, and treatment planning for various conditions such as tumors, infections, blood clots, and internal bleeding [9]. Compared to conventional X-rays, CT scans offer healthcare providers superior image resolution, comprehensively depicting diverse bodily structures. This enhanced clarity aids in the identification and assessment of diseases and injuries that may evade detection through traditional X-ray imaging [11]. CT scans often involve administering contrast agents to heighten the visibility of specific organs or tissues, facilitating a more accurate interpretation of the results [12]. The contrast material may be introduced orally, intravenously, or via an enema, augmenting the radiologist's capacity to discern abnormalities during the examination [13]. While CT scans are generally deemed safe and minimally invasive, they entail exposure to ionizing radiation, which carries a slight risk of short-term and long-term health implications [10]. Consequently, patients must engage in thorough discussions with their healthcare providers regarding the benefits and potential risks associated with CT scans, particularly if they harbor concerns regarding radiation exposure or if they are pregnant [10].

MRI

MRI is a noninvasive medical imaging modality that harnesses potent magnets and radio waves to generate intricate images of the body's internal structures. Unlike CT and positron emission tomography (PET) scans, MRI operates without the utilization of ionizing radiation, distinguishing it as a radiation-free imaging technique [14]. Widely employed across hospitals and clinics, MRI is a cornerstone in medical diagnosis, disease staging, and subsequent monitoring. Notably, MRI provides superior contrast in soft tissue imaging compared to CT scans, rendering it particularly invaluable for visualizing soft tissues such as those found in the brain or abdomen [15]. The methodology behind MRI involves aligning protons within the body with magnetic fields, followed by the emission of radio waves to perturb this alignment. Subsequently, the emitted signals as the protons realign are detected, culminating in the creation of detailed images depicting the body's internal structures [16]. MRI scans play a pivotal role in diagnosing an array of conditions, encompassing brain tumors, spinal cord issues, cardiac abnormalities, joint disorders, organ tumors, and breast cancer. Despite its efficacy, MRI may not be suitable for individuals with certain metal implants or electronic devices, owing to safety concerns associated with the powerful magnets utilized in the procedure [17]. Thus, careful consideration and evaluation are necessary to ensure the appropriateness of MRI imaging for each patient.

Challenges and limitations

**Figure 1:**
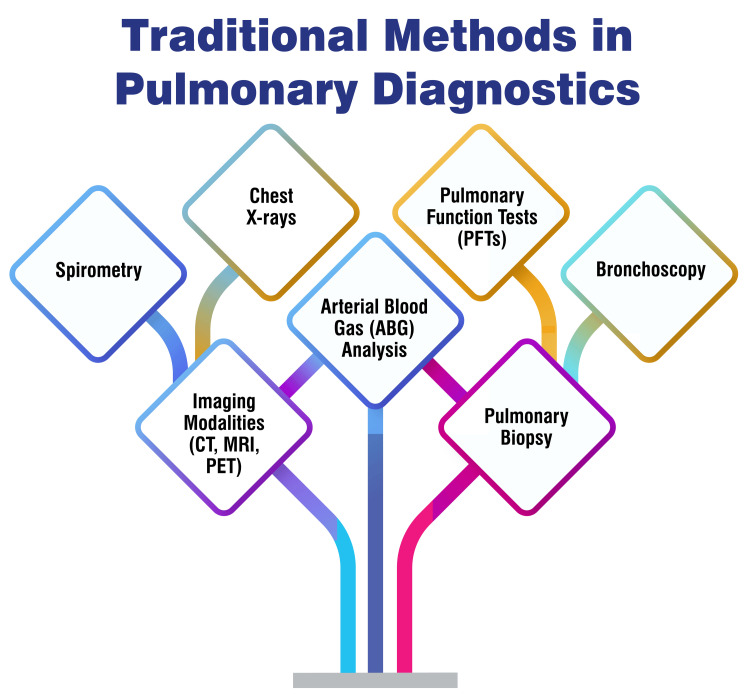
Traditional methods in pulmonary diagnostics The corresponding author Dr. Arman Sindhu self-created the figure.

The challenges and limitations inherent in the diagnosis and management of COPD represent a multifaceted issue that impacts healthcare systems globally. One significant challenge arises from the diverse clinical presentations of COPD, particularly in low- and middle-income countries (LMICs). In these regions, various clinical syndromes may be erroneously labelled as COPD due to the absence of reference values for spirometric criteria, potentially leading to misrepresentations of the disease [18]. Moreover, diagnosing COPD presents complexities, often compounded by the delayed onset of symptoms in the disease course. By the symptoms manifested, the disease may have progressed significantly, underscoring the critical importance of early detection. Early identification is pivotal for implementing interventions to modify risk factors such as tobacco use and to mitigate disease progression effectively [19]. Further challenges persist in differentiating between occupational and nonoccupational COPD. This delineation is particularly crucial for occupational COPD, where integrating new inflammatory markers for monitoring exposed workers and leveraging imaging techniques effectively pose ongoing diagnostic challenges [20]. Figure 1 shows traditional methods in pulmonary diagnostics.

Role of AI in lung imaging

Machine Learning Algorithms

Machine learning algorithms are pivotal in revolutionizing lung imaging and disease management, ushering in significant advancements in the field. These algorithms have been meticulously crafted to tackle various aspects of pulmonary health, including the identification of ILD in high-risk populations, the prediction of lung fibrosis severity, the correlation of radiological abnormalities, and the detection and classification of pulmonary nodules [21-23]. Using deep learning techniques, machine learning algorithms exhibit adaptability to diverse input data types, such as CT images featuring varying resolutions, noise levels, and contrast. This adaptability significantly bolsters their efficacy in accurately detecting lung nodules and predicting disease risks with precision [22]. Furthermore, these algorithms play a pivotal role in facilitating the early diagnosis of pulmonary nodules across different modalities, thereby enhancing the overall accuracy of lung cancer detection in PET/CT scans and aiding in the formulation of tailored treatment plans [22]. Integrating machine learning algorithms into lung imaging amplifies diagnostic capabilities and paves the way for personalized management strategies tailored to individual patients. By leveraging the insights gleaned from these algorithms, this symbiotic relationship between machine learning and lung imaging holds immense promise for the future of pulmonary healthcare, offering a beacon of hope for patients and clinicians alike.

Deep Learning Techniques

Deep learning techniques have spearheaded remarkable advancements in the realm of lung imaging, particularly in the diagnosis and management of lung cancer. These cutting-edge methodologies involve using sophisticated algorithms to scrutinize medical images, notably CT scans, for tasks such as pulmonary nodule detection, segmentation, and the differentiation between benign and malignant nodules [24]. Through the intricate analysis of these images, deep learning methods have exhibited remarkable efficacy in diagnosing lung cancer malignancy, furnishing accurate and expedited assessments of lung damage on a global scale, and leveraging the wealth of data available through CT scans [25]. Furthermore, deep learning has emerged as a formidable tool for medical image analysis, leveraging the automatic learning of feature representations directly from data. This innovative approach has catalyzed breakthroughs in medical imaging, elevating the landscape of image analysis in various lung pathologies, including pulmonary nodule diseases, pulmonary embolism, pneumonia, and ILD [26]. The application of deep learning techniques in pulmonary medical imaging has yielded significant enhancements in classification, detection, and segmentation tasks, heralding a promising avenue for a more precise and efficient diagnosis and treatment planning in a spectrum of lung disorders. This paradigm shift augments the diagnostic capabilities of healthcare providers and holds profound implications for optimizing patient care and outcomes in pulmonary medicine.

Natural Language Processing (NLP) for Radiology Reports

NLP for radiology reports entails the automated analysis of textual data within radiology reports to extract valuable insights for healthcare applications. Leveraging advanced NLP techniques, the unstructured text data is transformed into structured formats, facilitating the retrieval of detailed information from imaging reports without manual intervention [27-29]. This process expedites research, enhances healthcare quality improvement initiatives, and enables large-scale health data analysis by unlocking the wealth of patient health and disease information encapsulated within radiology reports. NLP in radiology reports assumes a pivotal role across various domains, including disease information extraction, diagnostic surveillance, and clinical decision support. Through applying NLP, researchers and healthcare professionals can efficiently pinpoint critical information such as disease classifications, diagnostic findings, and disease surveillance data embedded within radiology reports [27,29]. Furthermore, NLP aids in refining the accuracy and efficiency of clinical workflows by automating the extraction of structured data from free-text imaging reports. By harnessing the capabilities of NLP in radiology reports, stakeholders in the healthcare ecosystem stand to benefit from streamlined processes, enhanced data accessibility, and improved clinical decision-making. This transformative approach optimizes healthcare delivery and empowers professionals with actionable insights derived from radiological data, fostering improved patient care outcomes.

Computer-Aided Detection and Diagnosis (CAD)

CAD systems are pivotal tools in medical imaging, aiding radiologists in image interpretation and bolstering diagnostic precision. These systems harness many computer algorithms, including image processing techniques, classification systems, and AI methodologies such as artificial neural networks (ANNs) [30]. The scope of CAD applications has expanded across various imaging modalities, encompassing radiography, ultrasound, CT, mammography, MRI, and PET. CAD systems address a broad spectrum of diseases, spanning lung cancer, breast cancer, colon cancer, Alzheimer's disease, and beyond [30]. CAD systems are categorized into two primary groups: computer-aided detection (CADe) and computer-aided diagnosis (CADx). CADe systems concentrate on lesion localization within medical images, while CADx systems focus on lesion characterization, aiding the differentiation between benign and malignant tumors [31]. The overarching objective of these systems is to enhance radiologists' accuracy, streamline interpretation processes, and optimize decision-making workflows. For instance, CAD systems tailored for lung cancer have demonstrated promising outcomes in detecting and diagnosing pulmonary nodules on CT images, boasting high accuracy rates and sensitivity levels [32]. By augmenting radiological practices with advanced computational methodologies, CAD systems represent a cornerstone in modern medical imaging, propelling diagnostic capabilities to unprecedented heights.

Applications of AI in lung imaging

Early Detection of Lung Cancer

Developed collaboratively by researchers at the Mass General Cancer Center and the Massachusetts Institute of Technology, the Sybil AI tool has showcased remarkable accuracy in predicting an individual's likelihood of developing lung cancer within the subsequent year, with accuracy rates ranging from 86% to 94% [33]. This cutting-edge AI tool analyzes CT scans distinctively, capable of detecting subtle early signs of lung cancer that may elude detection by the human eye alone. As a result, it facilitates timely intervention and significantly enhances patient outcomes. In a separate study, researchers underscore the potential of AI-enhanced nanosensing technology for the early detection of lung cancer through the analysis of chromatin alterations within the field [34]. This pioneering approach harnesses the power of AI to augment optical nanosensing capabilities, presenting a promising avenue for identifying lung cancer at its incipient stages. By marrying advanced AI with nanotechnology, this innovative methodology holds immense promise in revolutionizing early cancer detection strategies.

Quantification of Lung Nodules and Lesions

The quantification of lung nodules and lesions represents a pivotal realm within AI applications in lung imaging. Advanced AI models, exemplified by the AI-rapid application development (RAD) platform, have been meticulously developed for automate lung nodular detection, localization, and segmentation utilizing chest CT scans [35]. These models undergo extensive training on a vast repository of manually curated scans to ensure precise identification and quantification of lung abnormalities. The AI-RAD platform stands out for its remarkable sensitivity in nodule detection, offering the flexibility to adjust parameters such as nodule size and number to optimize performance [35]. Despite its considerable success, challenges persist, notably in false-positive rates that may precipitate unnecessary patient procedures. To address this issue, ongoing research endeavors are imperative to refine these parameters and bolster the specificity of AI models in accurately quantifying lung nodules. Through continued refinement and validation, AI-driven quantification of lung nodules holds immense potential to enhance diagnostic accuracy and streamline patient care pathways. As researchers continue to unravel the complexities of lung imaging, the integration of AI technologies promises to usher in a new era of precision medicine, ultimately benefiting patients and healthcare providers alike.

Classification of Lung Diseases

The classification of lung diseases, including pneumonia and COPD, has witnessed substantial advancements by integrating AI and machine learning algorithms into medical imaging. Notably, studies have showcased the effectiveness of deep learning methodologies, particularly convolutional neural networks (CNNs), in accurately classifying various lung diseases based on distinctive features extracted from medical images such as X-rays and CT scans [36,37]. AI models have been intricately tailored to discern between specific lung pathologies, including COVID-19, pneumonia, and normal lung tissue, underscoring the versatility of AI in disease differentiation [37]. Furthermore, ongoing research endeavors have been dedicated to the development of AI architectures capable of multiclass classification of diverse lung diseases, encompassing pneumonia, lung cancer, tuberculosis (TB), lung opacity, and beyond, thereby exemplifying the expansive scope of AI applications in lung disease categorization [36]. The landscape of AI-empowered lung disease classification and detection continues to evolve, with concerted efforts directed toward overcoming challenges, enhancing accuracy, and exploring novel techniques such as deep learning, machine learning, and computer vision to augment disease classification capabilities [38]. Through collaborative interdisciplinary research and technological innovation, the integration of AI into lung disease classification holds immense promise for revolutionizing diagnostic paradigms and improving patient care outcomes in pulmonary medicine.

Predictive Modeling for Treatment Response and Prognosis

Several studies highlight the utility of predictive modeling across various medical domains. One such study focuses on personalized prediction of therapy effectiveness in relapsing-remitting multiple sclerosis (RRMS). Employing hierarchical Bayesian generalized linear models, this study estimates treatment response and evaluates model performance using various performance metrics [39]. Predictive modeling is also significant in cancer research, contributing to drug development, risk prediction, and treatment planning. These models enable anticipation of outcomes, facilitate clinical decision-making, and enhance cancer prevention strategies [40]. In the realm of oncology, predictive models have been developed utilizing both linear and nonlinear algorithms to forecast survival, nonlocal failure, radiation-induced liver disease, and other outcomes in patients with specific conditions such as metastatic castration-resistant prostate cancer [41].

**Figure 2:**
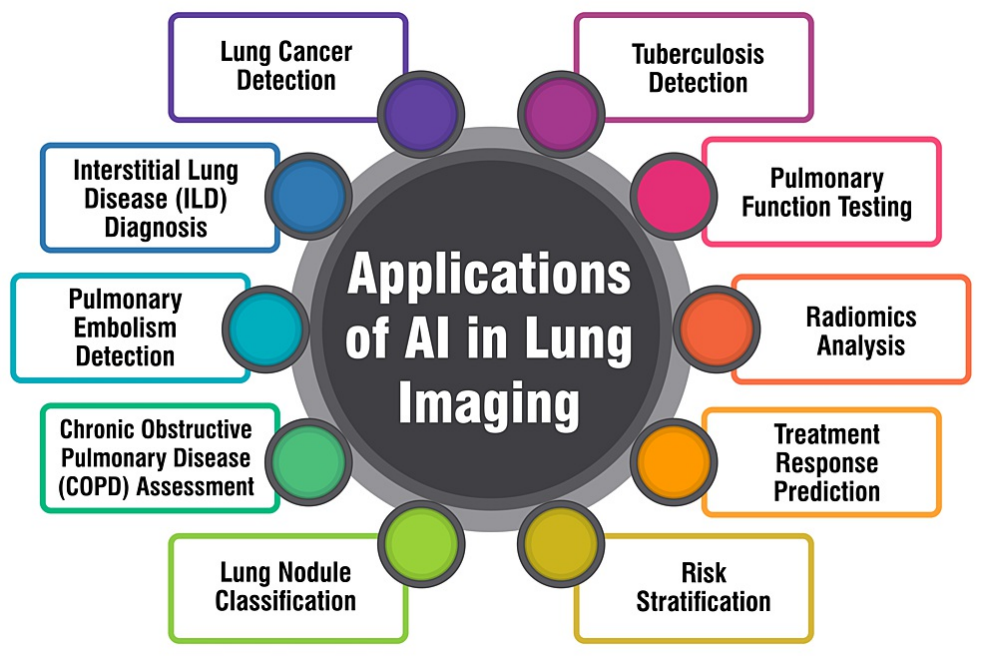
Applications of AI in lung imaging The corresponding author Dr. Arman Sindhu self-created the figure.

Moreover, predictive modeling is instrumental in understanding treatment response in diseases like B-cell acute lymphoblastic leukemia. A study delves into identifying factors influencing the clinical response of chimeric antigen receptor (CAR) T-cell therapy for refractory or relapsed B-cell acute lymphoblastic leukemia. Predictive models are constructed to estimate treatment response and provide valuable insights for accurate diagnosis and targeted treatment [42]. Furthermore, predictive models are indispensable in clinical care settings, emphasizing the conditional modeling of survival based on treatment received and other predictor variables. These models aim to assist individual patients and clinicians in selecting the most suitable treatments based on predicted outcomes, thereby optimizing patient care and treatment outcomes [43]. Figure 2 shows the applications of AI in lung imaging.

Case studies and examples

Success Stories of AI Implementation in Pulmonary Diagnostics

The success stories of AI implementation in pulmonary diagnostics exemplify AI's transformative impact on revolutionizing lung disease management. One notable achievement involves a research group at Nagoya University developing an AI algorithm capable of accurately and rapidly diagnosing idiopathic pulmonary fibrosis, a potentially life-threatening lung condition, with accuracy comparable to that of human specialists. This AI algorithm harnesses noninvasive examinations, including lung images and medical data, to enable early disease diagnosis, providing a vital tool for medical professionals to enhance diagnostic precision and obviate the need for invasive procedures such as lung biopsies [44]. Furthermore, recent advancements in AI applications in ILDs have showcased the potential of AI in screening, diagnosing, and categorizing various ILDs based on many parameters, including imaging modalities, genetic data, spirometry, and lung diffusion tests. AI tools have proven instrumental in analyzing intricate datasets about ILDs, facilitating accurate diagnosis, classification, and prognosis prediction. The integration of AI in ILDs represents a significant stride toward improving patient outcomes and streamlining clinical workflows in pulmonary medicine [45].

Comparative Analysis with Traditional Methods

In comparative analyses with traditional methodologies, deep learning techniques have displayed significant promise in enhancing the accuracy of lung nodule diagnosis. Research findings indicate that deep learning methods surpass traditional machine learning approaches for image processing, particularly in refining the accuracy of lung nodule diagnosis [22]. This underscores the superior capabilities of deep learning methodologies in tackling intricate imaging tasks associated with lung nodules. Furthermore, a study juxtaposing the diagnostic efficacy of traditional FlyerScan and deep learning-based medical open network for AI (MONAI) adult CT imaging techniques in children revealed notably diminished diagnostic performance for both methodologies [46]. This comparison underscores the potential limitations of conventional imaging techniques when applied to pediatric populations. It accentuates the necessity for advanced AI-driven approaches to bolster diagnostic accuracy in specific patient cohorts.

Challenges Faced and Lessons Learned

Developing AI algorithms for medical imaging encounters significant challenges due to the complexity of medical imaging data and patient privacy concerns [47]. Furthermore, data collection and interpretation biases can skew results, impacting the accuracy and reliability of AI models [48]. It is imperative to distinguish between causation and correlation in AI applications to avoid drawing misleading conclusions based solely on predictive power [48]. Ensuring the integrity of AI models is vital to prevent cheating or manipulation that could compromise diagnostic accuracy and patient outcomes [48]. Access to diverse and comprehensive medical data is necessary for developing and validating AI algorithms for medical imaging applications [47]. Collaborating with subject matter experts is crucial to effectively address complex medical challenges by combining AI expertise with domain-specific knowledge [48]. Rigorous validation and testing processes are essential to guarantee the reliability and generalizability of AI algorithms in medical imaging applications [49]. Fostering a culture of continuous learning and improvement is essential for overcoming challenges and optimizing the integration of AI in healthcare settings [50]. Addressing ethical considerations, such as bias mitigation, privacy protection, and transparency, is fundamental to deploying AI solutions responsibly in medical imaging [49]. Leveraging expertise from various disciplines, including radiology, computer science, and medical physics, enhances the development and implementation of AI technologies in medical imaging [50].

Future directions and challenges

Integration of AI into Clinical Workflows

Integrating AI into clinical workflows represents a transformative process with significant benefits in healthcare settings. At Lahey Hospital & Medical Center, radiologists have successfully incorporated AI algorithms into their clinical workflow to diagnose and triage imaging studies for critical findings, thereby enhancing patient care [51]. These AI algorithms play a crucial role in prioritizing potentially positive studies, such as those indicating pulmonary embolisms or intracranial hemorrhages, by moving them to the top of the worklist. Reducing radiologists' workload and improving efficiency contribute to the swift identification of urgent cases. The adoption of AI in clinical workflows necessitates a thoughtful approach, as emphasized by experts advocating for measured integration to ensure optimal outcomes. The American College of Radiology's Data Science Institute has developed AI Central™. This platform offers access to FDA-approved algorithms and supporting materials to assist practices in selecting the most suitable AI tools tailored to their specific goals [52]. This strategic adoption process entails identifying use cases for AI, comparing vendors through platforms like AI Central™, and collaborating with vendors to seamlessly integrate AI algorithms into existing workflows, enhancing radiologists' usability [53].

Ethical Considerations and Patient Privacy

In AI applications in healthcare, ethical considerations and patient privacy assume paramount importance, particularly within lung imaging. The ethical framework governing medical AI underscores the significance of data quality, algorithmic bias, transparency, safety, security, and responsibility attribution to ensure the trustworthiness of AI systems [54]. In medical AI applications, patient privacy protection emerges as a critical ethical concern, especially concerning lung cancer image data and the utilization of AI in radiology. Obtaining informed consent for AI systems is imperative, necessitating clear communication regarding the purpose and potential implications of AI usage to uphold patient autonomy and privacy [55,56]. Furthermore, ethical deliberations encompass data ownership, consent for data reuse, and safeguarding sensitive patient data, all pivotal in AI-driven healthcare environments. Regulations like the EU General Data Protection Regulation (GDPR) mandate explicit patient consent for data sharing and reuse in AI algorithm training, emphasizing the ethical imperative of safeguarding patient privacy [55].

Regulatory Approval and Adoption by Healthcare Institutions

Regulatory approval and adoption by healthcare institutions of AI applications, particularly in medical imaging, are pivotal factors that influence the integration of AI into healthcare systems. The regulatory frameworks governing software as medical device applications play a crucial role in ensuring the safety and effectiveness of AI-based diagnostic imaging algorithms. However, current regulatory frameworks exhibit significant gaps, including the conflation of the diagnostic task with the diagnostic algorithm, superficial treatment of diagnostic task definition, and the absence of mechanisms to compare similar algorithms [57,58] directly. Healthcare institutions need help adopting AI, stemming from regulatory barriers, limitations in data access, and misaligned incentives. The sluggish adoption of AI in healthcare can be attributed to privacy regulations impeding data collection, protracted regulatory approval processes, and concerns regarding liability in the event of AI failures leading to malpractice lawsuits [58,59]. Addressing these challenges necessitates complementary innovations in regulation, data-sharing practices, and liability frameworks to facilitate the safe and effective adoption of AI technologies in healthcare settings.

Emerging Trends and Potential Advancements

**Figure 3:**
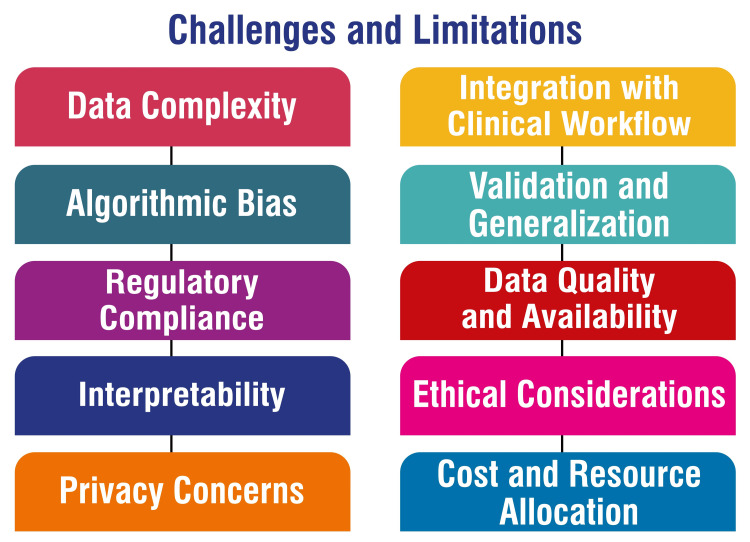
Challenges and limitations The corresponding author Dr. Arman Sindhu self-created the figure.

The future of healthcare is being shaped by emerging trends and potential advancements in AI applications in lung imaging. Recent progress underscores the utilization of AI in ILDs, focusing on screening, diagnosis, and prognosis. Key data repositories such as the Open Source Imaging Consortium (OSIC) data repository, ILD Database from medGIFT, ILDgenDB, and ILDGDB play a pivotal role in facilitating research and development efforts aimed at predicting lung function decline and unraveling gene mechanisms associated with ILDs [45]. A noteworthy development is the creation of AI tools like Sybil, which are engineered to detect early signs of lung cancer that may elude detection by the naked eye on CT scans. Sybil's remarkable ability to predict an individual's risk of developing lung cancer within the next year with high accuracy underscores the potential for AI to revolutionize early cancer detection. This breakthrough has the potential to significantly impact patient outcomes by enabling earlier intervention and treatment for lung cancer, ultimately enhancing survival rates [33]. Looking ahead, AI in lung imaging is poised for further expansion with advancements in multimodal models integrating patient data streams, foundational AI models trained on extensive datasets, and specialized medical AI models tailored for specific conditions such as lung cancer. However, addressing challenges such as regulation, quality assurance, data diversity, and transparency in AI algorithms will be critical for maximizing the potential benefits of AI in lung imaging while safeguarding patient safety and ensuring equitable access to advanced healthcare technologies [60-62]. Figure 3 shows challenges and limitations.

## Conclusions

In conclusion, integrating AI into pulmonary diagnostics has heralded a transformative shift in the field, promising a more accurate and efficient assessment of lung health. Throughout this review, we have delineated the myriad applications of AI, spanning from early detection of lung cancer to predictive modeling for treatment response. The impact of AI on revolutionizing pulmonary diagnostics cannot be overstated, as it enables healthcare professionals to make more informed decisions, leading to improved patient outcomes. However, it is crucial to acknowledge the challenges associated with AI implementation, including ethical considerations, regulatory hurdles, and the need for ongoing research. Collaborating efforts among healthcare professionals, researchers, and industry stakeholders are imperative to validate AI algorithms, optimize their performance, and ensure seamless integration into clinical practice. Moreover, continued investment in AI research and development will be pivotal in unlocking its full potential and enhancing pulmonary healthcare globally. By fostering interdisciplinary collaboration and innovation, we can leverage the power of AI to revolutionize pulmonary diagnostics and ultimately improve patients' lives worldwide.
